# Cromolyn inhibits the secretion of inflammatory cytokines by human microglia (HMC3)

**DOI:** 10.1038/s41598-021-85702-8

**Published:** 2021-04-13

**Authors:** Yi-Jun Wang, Alina Monteagudo, Matthew A. Downey, Philip G. Ashton-Rickardt, David R. Elmaleh

**Affiliations:** 1 AZTherapies Inc., Boston, MA USA; 2 Department of Radiology, Massachusetts General Hospital, Harvard Medical School, Boston, MA 02129-2060 USA

**Keywords:** Chemokines, Cytokines, Inflammation, Neuroimmunology, Alzheimer's disease, Dementia, Neurodegeneration, Neurodegenerative diseases, Microglial cells

## Abstract

Cromolyn is a known mast cell stabilizer and is approved for treatment of asthma and for other allergic indications. Cromolyn, in a new redesigned dry powder formulation, is being tested in a pivotal clinical trial in combination with low dose ibuprofen to treat early Alzheimer’s Disease (AD) subjects. To better understand the mechanistic effect cromolyn has in slowing down or halting the neuroinflammatory response associated with AD progression, we tested the effect of cromolyn to dampen the inflammatory response in the human HMC3 microglia cell line. The direct effect of cromolyn on HMC3 microglia is on cytokines and chemokines production following their activation by the inflammatory cytokine TNF-α. Cromolyn and a new fluorinated analog dramatically reduced the secretion of a wide spectrum of inflammatory mediators, which included cytokines such as IL-1β, IL-6, IL-8 and IFN-γ, and chemokines such as CXCL10, CCL2, CCL3 and CCL4. These results bolster our understanding of how our cromolyn platform modulates toxic microglia behavior as a dynamic future treatment option for neurodegenerative disorders.

## Introduction

Microglia are the resident macrophages of the central nervous system (CNS) and have critical roles with immune surveillance, maintenance of synapses, and neuronal health^1^. Microglia are distinct from other myeloid cells, such as monocytes and peripheral macrophages, and are extremely reactive to changes within the brain, collectively existing as heterogeneous populations determined by the homeostatic requirements of a given region of the CNS that respond to extracellular changes to protect from infection^2–4^. Microglia mediate these important and diverse functions through the secretion of cytokines, chemokines as well as growth and neurotrophic factors^5^. In addition, microglia exhibit direct anti-microbial activity through phagocytosis and the production of microbicidal Reactive Oxygen Species (ROS) and nitric oxide (NO)^6^.

The prominent roles microglia have in regulating inflammation are usually designated by one of two phenotypes: the M1-type where microglia produce pro-inflammatory, neurotoxic cytokines whose chronic production contributes to dysfunction of the neural network and propagates inflammation reaction; or the M2 type where microglia secrete anti-inflammatory mediators and neurotrophic factors that are involved in restoring brain homeostasis^6^. Simplifying microglia into only two designations makes it simpler to refer to a generalized activation state of microglia, but it is more likely that microglia exist on a dynamic spectrum that activate specifically in response to chronic stressors that lead to neurodegeneration in the CNS^7^.

It is generally accepted that early activation of monocytes and microglia to the M2 phenotype has potential to decelerate neurodegenerative progression by modulating immune responses without triggering secretion of pro-inflammatory cytokines that could worsen neurodegeneration^8^. Strategies to modulate monocyte and microglial activity have been studied, especially those that can protect against microglia-mediated neurotoxicity^9–11^. We are focused on studies that would establish the utility of our cromolyn analogs as a platform to modulate the pathology of neurodegenerative diseases such as Alzheimer’s Disease (AD) and Amyotrophic Lateral Sclerosis (ALS). The role of the neuro-inflammatory response in the presence of amyloid plaques and neurofibrillary tangles in the brain and its associated neuronal loss in the pathology of AD is well established and extensively studied^12–15^. Numerous studies show that microglial-mediated inflammation contributes to the progression of AD and that microglial cells are found in close association with amyloid-β (Aβ) deposits^16^.

Cromolyn is a small molecule approved for the treatment of asthma^17^. Although the molecular mechanism of action is not well understood, cromolyn has been known for some time as a “mast cell stabilizer”^18^ and to have other anti-inflammatory effects on polymorphonuclear neutrophil (PMN)^19^ and macrophages^20^. Screening of a pharmaceutical library of compounds that have reached clinical trial stages in the US identified cromolyn as having potential to reduce Aβ42:Aβ40 burden in human iPSC-derived neurons by 20% and by more than 50% in combination with topiramate and bromocriptine^21^. This independently verifies the in vivo cromolyn study by Hori et al., that reduced soluble Aβ by 50% in one week and showed alterations in microglia economy^22^. More recently, cromolyn has been shown to promote the non-inflammatory phagocytosis of Aβ42 by murine BV2 microglial cells as well as improved clearance of TBS-soluble Aβ40 and Aβ42 and improved neuroprotection in Tg2576 AD model mice^10^. Furthermore, cromolyn reduced the levels of pro-inflammatory cytokines and mast cell activity in SOD1 transgenic mouse models from ALS^23^.
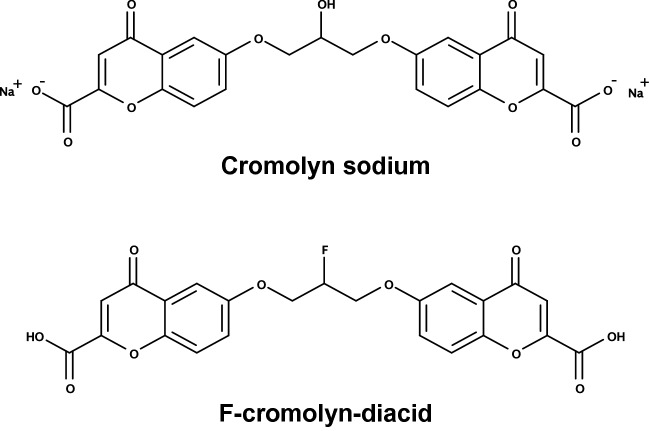


Our group has provided pre-clinical evidence for the role of cromolyn in the treatment in APP/PS1 mice as an animal model of AD^10,22^. On this basis, cromolyn is currently under evaluation in a late-stage clinical trials for early onset AD (NCT02547818—A Phase III Safety and Efficacy Study of ALZT-OP1 in Subjects with Evidence of Early Alzheimer’s Disease) and ALS (NCT04428775—A Phase II Safety and Biomarker Study of ALZT-OP1a in Subjects with Mild-Moderate ALS Disease). To determine whether cromolyn inhibits secretion of pro-inflammatory mediators by microglia, we examined the effect of cromolyn on the HMC3 microglial cell line^24^ after stimulation with TNF-α. Cromolyn dramatically reduced the secretion of a wide spectrum of inflammatory mediators, which included cytokines such as IL-1β, IL-6, IL-8 and IFN-γ, and chemokines such as CXCL10, CCL2, CCL3 and CCL4. Experiments in our group to modify the cromolyn structure to increase lipophilicity and blood–brain barrier uptake resulted in substitution of the bridge hydroxyl group for fluorine to produce F-cromolyn^25^. In this study we show that the core cromolyn structural motif was able to impact cytokine and chemokine release by activated human HMC3 microglia and these benefits are retained with the modified F-cromolyn. These results expand the robust cromolyn platform to include inhibition of inflammatory mediators secreted by microglia, further bolstering cromolyn as an exciting therapeutic to combat neurodegeneration.

## Materials and methods

### Chemicals and reagents

DMEM (Dulbecco’s Modified Eagle Medium) (Cat#1995065), DMEM without phenol red (Cat#21063029), PBS-pH7.2 (Cat#20012050), L-glutamine (Cat#25030081), Trypsin–EDTA (Cat#25200056), and penicillin–streptomycin (Cat#15140122) were products of Gibco, Thermo Fisher Scientific. FBS (fetal bovine serum) (Cat#F4135), DMSO (dimethyl sulfoxide) (Cat#D2438), and TWEEN-20 (Cat#P1379) were purchased from Sigma-Aldrich. 3 mL Syringe/Needle Combination with Luer-Lok™ Tip (Cat#8936G82), 13 mm syringe filter (PVDF, 0.22 µm) (Cat#1159T77) were purchased from Thomas Scientific. Recombinant human TNF-α (Cat#300-01A), recombinant human IFN-γ (Cat#300-02) were purchased from PeproTech. Cromolyn and F-cromolyn were provided by AZTherapies and DMEM was used as diluent to achieve final concentrations as indicated.

### Cell line and cell culture

Human microglial cell line HMC3 (CRL-3304) was purchased from ATCC (American Type Culture Collection). HMC3 cell line was cultured in DMEM medium with 10% FBS, 1% L-glutamine, and 1% penicillin/streptomycin. Cells were maintained in 37 °C incubator at 5% CO_2_.

### Flow cytometry

HMC3 cells were resuspended, counted using the LUNA-II Automated Cell Counter, seeded in the 6-well plate (300 K cells/2 mL medium/well), and incubated overnight to allow cells to attach. Cells were treated with TNF-α or IFN-γ at five different concentrations (0, 0.01, 0.03, 0.1, 0.3 µg/mL) for 24 h to induce cell activation. The supernatant medium was completely aspirated and 1 mL of fresh DMEM was added to wash the cell layer to remove detached cells and debris. The attached cells were resuspended and washed with PBS. Cells were washed with FACS buffer (2% FBS in PBS) prior to incubation with Zombie Violet Fixable Viability Dye (#423114, Biolegend, 1:500 dilution) and Human Fc Receptor Blocking Solution (#422302, Biolegend, 1:20) diluted in FACS buffer at RT for 10 min in the dark. Cells were then washed with FACS buffer and incubated at RT for 20 min with the following cell-surface antibodies: FITC anti-MHC II (#361706, Biolegend), PerCP/Cy5.5 anti-CD11b (#301328, Biolegend), BV605 anti-CD40 (#334336, Biolegend), PE anti-CD86 (#374206, Biolegend), PE/Cy7 anti-CD163 (#326514, Biolegend), APC anti-CD206 (#321110, Biolegend), and PerCP/Cy5.5 anti-CD14 (#367110, Biolegend). All of the cell-surface antibodies were diluted 1:20 in FACS buffer. Cells were permeabilized using the Transcription Factor Fixation/Permeabilization Buffer Set (#424401, Biolegend). Then cells were incubated with the following intracellular antibodies: APC/Cy7 anti-CD68 (#333822, Biolegend), purified anti-IBA1 (#PA5-27436, Invitrogen), Goat anti-Rabbit-Alexa Fluor 647 (#A21245, Invitrogen), purified anti-GFAP (#644702, Biolegend), and Goat anti-Mouse-Alexa Fluor 488 (#11029, Invitrogen). All of the intracellular antibodies were diluted 1:100 in the Permeabilization buffer (1x). Cells were washed with Permeabilization buffer and fixed in Fixation Buffer (#420801, Biolegend). Flow cytometry was performed using the Attune NxT Acoustic Focusing Cytometer (Thermo Fisher Scientific). Analysis of flow cytometry data was performed via FCS Express 7 (DeNovo Software). All flow cytometric diagrams are provided in Supplementary Figure S1.

### Cell viability assay by AO/PI stain

Cell viability was assessed with a LUNA-FL Automated fluorescence cell counter. Viable nucleated cells show green fluorescence and dying nucleated cells show red fluorescence. 18 μL of sample was mixed with 2 μL Acridine Orange/Propidium Iodide (AO/PI) Stain.

### MSD U-PLEX assay platform—cytokine and chemokine secretions

HMC3 cells were resuspended, counted using the LUNA-II Automated Cell Counter, seeded in the 6-well plate (400 K cells/2 mL medium/well), and incubated overnight to allow cells to attach. The media and detached cells were removed next day. The cell layer was washed three times in PBS and once in serum- and phenolred-free DMEM (SPFM). Cells were incubated in SPFM for 4 h prior to treatment with TNF-α (0.3 µg/mL) and/or Cromolyn (0.3 µM, 3 µM) or F-cromolyn-diacid (0.3 µM, 3 µM) for 24 h. The conditioned medium was collected and centrifuged at 1000 × RCF (g) for 5 min to pellet detached cells and large debris, which was subsequently passed through a 0.22 µm filter with PVDF membrane to remove smaller debris. Samples of the supernatant medium were put in a CoolRack (#07210041, Fisher Scientific) on dry ice for Snap-freezing and kept at − 80 ºC until use.

Meso-scale U-PLEX plates that detect a cytokine panel including IL-1β, IL-2, IL-4, IL-6, IL-8, IL-10, IFN-γ, TNF-α, and a chemokine panel including IP-10/CXCL10, MCP-1/CCL2, MIP-1α/CCL3, MIP-1β/CCL4, Eotaxin/CCL11, were used as per manufacture’s protocol. 25 µL of the conditioned medium was used in each well of the MSD plates to detect the analytes. The plates were then analyzed on an MSD QuickPlex SQ120 instrument. All standard concentration curves for cytokines and chemokines tested in this study are provided in the Supplementary Figure S3.

### Reverse transcription (RT) and quantitative PCR

Total RNA was prepared from cells using the Quick-RNA MiniPrep kit (Zymo Research), according to the manufacturer’s instructions. cDNA was prepared from total RNA using High-Capacity cDNA Reverse Transcription Kit (Thermo). Following reverse transcription, quantitative PCR was performed using a cycling profile consisting of 95 °C for 2 min (Stage I), 40 cycles of 95 °C for 20 s, 60 °C for 30 s, and 70 °C for 30 s (Stage II), and 65 °C for 5 s (Stage III). Fold changes were determined by subtracting Cq values of the loading control GAPDH from the Cq values of the gene of interest. The results were normalized to untreated controls. Primers used for qRT-PCR are listed as follows: human GAPDH-F: ACAACTTTGGTATCGTGGAAGG; human GAPDH-R: GCCATCACGCCACAGTTTC; human IL6-F: ACTCACCTCTTCAGAACGAATTG; human IL6-R: CCATCTTTGGAAGGTTCAGGTTG; human IL8-F: ACTGAGAGTGATTGAGAGTGGAC; human IL8-R: AACCCTCTGCACCCAGTTTTC; human CD11b (ITGAM)-F: ACTTGCAGTGAGAACACGTATG; human CD11b (ITGAM)-R: TCATCCGCCGAAAGTCATGTG; human TMEM119-F: CGGCCTATTACCCATCGTCC; human TMEM119-R: CTGGGCTAACAAGAGAGACCC; human GFAP-F: CTGCGGCTCGATCAACTCA; human GFAP-R: TCCAGCGACTCAATCTTCCTC.

### Quantification and statistical analysis

All the data were presented as mean ± standard error from at least three times, each done in triplicate. The statistical significance between two groups was determined by Student’s t test, whereas the comparisons of multiple groups were carried out by one-way ANOVA, followed by Bonferroni’s post-test using GraphPad Prism 7 (GraphPad Software, Inc.). A probability value of * *p* < 0.05 was considered to be significant.

## Results

### F-cromolyn justification

A significant hurtle to overcome when treating CNS disorders is delivery of a given drug into the CNS while maintaining in vivo stability and subsequent drug activity. Fluorine substitutions are utilized in medicinal chemistry to increase important pharmacokinetic properties of drugs such as blood–brain barrier permeability, metabolic stability, and bioavailability^26^. C–F bonds have higher bond energies than C–H and C–O bonds and thus greater stability against proteolytic degradation and increased relative lipophilicity^26^. The log P of compound is a measure of its lipophilicity and is associated with its ability to cross the blood–brain barrier, and the substitution of hydrogen and other functional groups for fluorine is known to modulate a compound’s log P^27^. As the hydroxyl group is considered a bioisostere of fluorine, with C–F and C–O bonds having similar bond length and isoelectronic properties^28^, the fluorine-substituted F-cromolyn compound in our experiments is expected to have increased metabolic stability and a lipophilicity for better uptake across cell membranes and more attuned to crossing the blood–brain barrier into the CNS, making F-cromolyn potentially useful as an orally available drug^25^.

### HMC3 microglia exhibit inflammatory phenotype after TNF-α exposure

Mast cells are an integral component of the innate immune system and inflammatory process in the skin and mucosal surfaces of the body but are also prevalent in the human brain^29^. Once matured, their degranulation and subsequent release of cytokines and other pro-inflammatory mediators and attractants initiate complex and robust responses to help eliminate infection. In the case of an asthmatic, allergic immune response, mast cell degranulation can be dampened with the inhaled use of cromolyn to alleviate an asthmatic attack^17^. Inflammation is a predominant characteristic of aging and neurodegenerative disorders^30^ and genetic mutations in microglial proteins such as TREM2 that have regulatory roles in microglial inflammation^31^ and others are risk factors for developing AD^32^.

TNF-α is an important inflammatory cytokine responsible for mediating and sustaining an inflammatory response in many cell types, including microglia, and clinical evidence has brought to light associations between TNF-α, aging, and Alzheimer’s disease. One longitudinal study found annual plasma TNF-α concentrations from 424 cognitively-normal, aging adults to be significantly increased over time and correlated with anatomical changes in the brain including gray matter volume and white matter hyperintensities burden, suggesting peripheral TNF-α may be mechanistically important in age-related decline^33^. Serum concentrations of TNF-α are also found to be upregulated in AD and in MCI^34^, and increased TNF-α serum levels have an associated four-fold greater rate of cognitive decline than AD patients with low baseline TNF-α^35^. Elevated cerebrospinal fluid (CSF) levels of TNF-α have positively correlated with *APOE4* status and decline in functional connectivity between regions of the brain involving memory, language, and higher decision making^36^. Collectively, these studies suggest an enduring link between high serum and CSF TNF-α levels, inflammation, and progressive cognitive decline in AD. We therefore sought to determine the ability of our cromolyn platform to dampen the secretion of inflammatory mediators in TNF-α induced HMC3 human microglia that behave similarly to microglia in the CNS.

**Figure 1:**
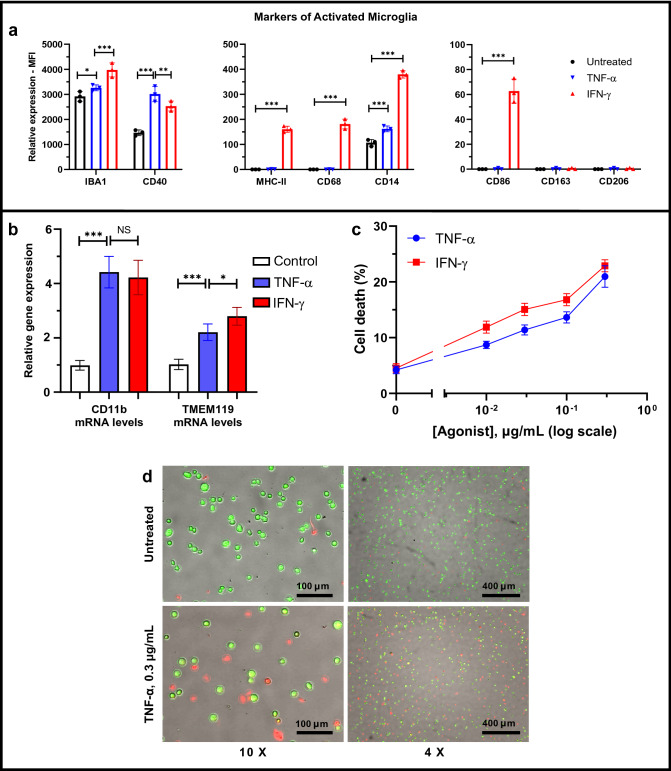
Inflammatory activation of HMC3 human microglia cell line by TNF-α and IFN-γ. (**a**) Flow cytometric analyses demonstrate the increased protein expression of inflammatory microglia biomarkers (IBA-1, CD40, MHC-II, CD68, CD14, and CD86) in the HMC3 cells treated with IFN-γ (0.3 µg/mL, 24 h) (red), but no alteration in the expression of the anti-inflammatory microglia biomarkers CD163 and CD206. TNF-α (0.3 µg/mL, 24 h) (blue) also increases the expression of inflammatory microglia biomarkers (IBA-1, CD40, and CD14). (**b**) q-PCR analysis revealed increased CD11b and TMEM119 expression by HMC3 microglia after addition of 0.3 µg/mL TNF-α or 0.3 µg/mL IFN-γ for 24 h. Fold change is normalized to untreated control. GAPDH was used as an internal control to normalize mRNA level of the gene of measurement in each sample (n = 5 independent experiments). Quantitative analysis (**c**) and representative fluorescence micrographs (**d**, displayed at ×10 and ×4 magnification) of the cell viability assay show that both TNF-α and IFN-γ induce cell death in a concentration-dependent manner (0, 0.01, 0.03, 0.1, 0.3 µg/mL) for 24 h in the HMC3 human microglial cells. Fluorescent green features are AO stains of live cells and fluorescent red features are PI stains of dying cells. *p < 0.1, **p < 0.01, ***p < 0.001, and NS (no significant difference).

The human microglial clone 3 (HMC3) microglia cell line was developed through SV40 immortalization of human primary microglial cells and is capable of reacting to inflammatory stimuli with regulation of typical markers of microglia activation^24^. The density of microglia is not uniform in all regions of the brain and the presence of surface markers for activated microglia differ depending on their location in the CNS^37^. As such, a microglial model of neuroinflammation need not exhibit all possible biomarkers for inflammation, though common biomarkers for activated microglia of an M1-like inflammatory phenotype include CD40, CD86, MHC-II, IBA-1, CD11b, CD14, and CD68^37^, all of which in our experiments, except in the case of CD11b that was not at first detected (data not shown), are increased after IFN-γ administration (Fig. 1a and Supplementary Figure S1). Despite this, we confirmed by q-PCR that mRNA expression levels of microglia markers CD11b and TMEM119 were present but modestly upregulated relative to controls after the addition of TNF-α and IFN-γ (Fig. 1b). Generalized surface markers of microglia such as CD11b and IBA-1 are found inconsistently upregulated in multiple brain regions of AD patients, but MHC-II and CD68 activation markers are more consistently increased in AD^38^. Interestingly, we observe expression of the astrocyte biomarker glial fibrillary acidic protein (GFAP) in resting, untreated HMC3 microglia and subsequent increase in GFAP after TNF-α and IFN-γ treatment, respectively, and confirm this result through q-PCR (Supplementary Figure S2). To our knowledge GFAP has not been reported to be expressed in HMC3 cells, but others have found microglia to be morphologically dynamic and can exhibit classical astrocyte markers from a variety of injurious stimuli^39–41^. The anti-inflammatory phagocytic surface markers CD163 and CD206 remained unexpressed and unchanged prior to and after the addition of both IFN-γ and TNF-α (Fig. 1a and Supplementary Figure S1). Altogether, it is reasonable that HMC3 microglia behave as expected for a model of neuroinflammation^24^.

We verified through cell viability experiments that both TNF-α and IFN-γ exert microglia necrosis in a concentration-dependent manner up to 0.3 µg/mL for each (Fig. 1c). Representative AO/PI fluorescence images for 0.3 µg/mL TNF-α are presented at 4× and 10× magnification in Fig. 1d. Interestingly, we observed a more robust range of cytokine and chemokine secretion in response to TNF-α than with IFN-γ stimulation (data not shown). This aspect is partially due to TNF-α inducing less cell death than IFN-γ at the same concentration and thus TNF-α was subsequently used to induce cytokine and chemokine secretion by HMC3 microglia prior to addition of cromolyn and F-cromolyn.

### Cromolyn and F-cromolyn inhibit secretion of inflammatory cytokines and chemokines from HMC3 human microglial cells

**Figure 2:**
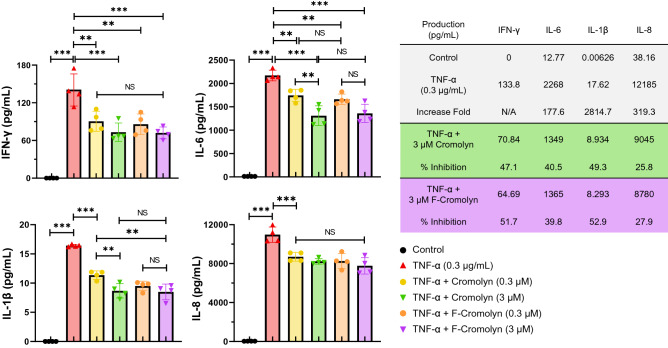
Cromolyn and F-cromolyn inhibit secretion of pro-inflammatory cytokines by HMC3 microglia cell line induced by TNF-α. Quantitative analyses of MSD assays indicate the dramatically increased secretion of IFN-γ, IL-1β, IL-6 and IL-8 from HMC3 cells treated with TNF-α (0.3 µg/mL, 24 h), which are subsequently reduced by cromolyn and F-cromolyn (0.3 µM and 3 µM for each). *p < 0.05, **p < 0.01, ***p < 0.001, and NS (no significant difference).

The cytokine TNF-α is known to be amply produced by monocytic cells, including microglia, and strongly influences many other inflammatory mediators to be upregulated by innate immune cells upon TNF-α exposure^42^. Our experiments verify that treatment of HMC3 cells with TNF-α potently induces inflammatory cytokine secretions by HMC3 cells as evidenced by the dramatic increase in cytokine concentration in cell culture supernatant after 24 h of TNF-α treatment (Fig. 2). Compared to untreated HMC3 cells (control), treatment with TNF-α (0.3 μg/mL) resulted in > 177-fold increase in IL-1β (*p* < 0.001), IL-6 (*p* < 0.001), and IL-8 (*p* < 0.001) and caused the previously quiescent cells to secrete IFN-γ. We verified that cromolyn and F-cromolyn do not affect the inflammatory profile of quiescent microglia and provide all cytokine and chemokine secretion data after addition of cromolyn and F-cromolyn to HMC3 microglia not induced by TNF-α in Supplementary Figure S4 as an internal control. We then tested the effect of both cromolyn and F-cromolyn at a low (0.3 µM) and higher (3 µM) concentration against TNF-α induced cytokine secretion by HMC3 microglia. IFN-γ (*p* = 0.0033; *p* = 0.0013), IL-6 (*p* = 0.0065; *p* = 0.0012), IL-1β (*p* < 0.001; *p* < 0.001), and IL-8 (*p* = 0.0006; *p* < 0.001) concentrations were decreased after addition of the lower 0.3 µM concentration of cromolyn and F-cromolyn, respectively, with only cromolyn dose-dependently reducing IL-6 (*p* < 0.001) and IL-1β (*p* < 0.001) after 3 µM addition. The higher 3 µM concentration of cromolyn resulted in > 40% inhibition of IFN-γ, IL-6, and IL-1β, and 25.8% inhibition of IL-8. F-cromolyn also decreased cytokine release at the higher 3 µM concentration, achieving > 50% inhibition of both IFN-γ and IL-1β and 39.8% and 27.9% inhibition of IL-6 and IL-8, respectively.

**Figure 3:**
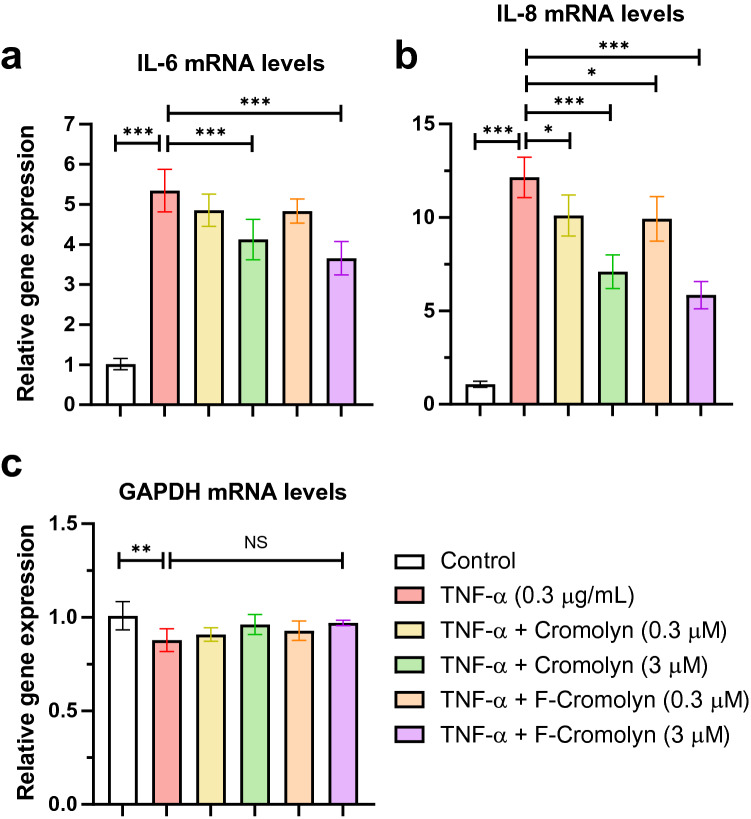
Cromolyn and F-cromolyn decrease the gene expression levels of IL-6 and IL-8 induced by TNF-α in HMC3 human microglia. Quantitative analyses of qRT-PCR assays show the significantly increased gene expression levels of (**a**) IL-6 and (**b**) IL-8 in HMC3 cells treated with TNF-α (0.3 μg/mL, 24 h), which are subsequently decreased by Cromolyn and F-cromolyn (0.3 μM and 3 μM) in a dose-dependent manner. Fold change is normalized to untreated control. (**c**) GAPDH was used as an internal control to normalize mRNA level of the gene of measurement in each sample. NS (no significant difference), *p < 0.05, **p < 0.01, ***p < 0.001, and n = 5 independent experiments.

To address whether cromolyn and F-cromolyn affected the gene expression of key inflammatory cytokines in HMC3 microglia, we performed qRT-PCR experiments to measure IL-6 and IL-8 mRNA expression (Fig. 3). We observed that relative to untreated controls, IL-6 expression increased approximately five-fold after addition of TNF-α (Fig. 3a). Upon addition of either 0.3 µM cromolyn or 0.3 µM F-cromolyn, there was a modest decrease in mRNA levels of IL-6 (9.0% and 9.5%, respectively, relative to TNF-α induced levels); however, increasing the concentration to 3 µM for both cromolyn and F-cromolyn significantly decreased IL-6 expression relative to TNF-α alone (*p* < 0.001; 22.8% and 31.6%, respectively, relative to TNF-α induced levels). Next, we observed an approximately 12-fold increase in mRNA expression of IL-8 after addition of TNF-α relative to untreated controls (Fig. 3b). IL-8 mRNA levels decreased after addition of 0.3 µM cromolyn and 0.3 µM F-cromolyn (*p* = 0.0212 at 16.7% and *p* = 0.0102 at 18.3%, respectively, relative to TNF-α induced levels) and decreased even further with the addition of 3 µM cromolyn and 3 µM F-cromolyn (*p* < 0.001; 41.5% and 51.9%, respectively, relative to TNF-α induced levels). We also analyzed GAPDH mRNA levels as an internal control^43^ to normalize total mRNA and cell protein levels over the same experimental conditions (Fig. 3c). Because GAPDH expression levels did not significantly change over any treatment group after TNF-α exposure, we interpret that decreases in cell numbers are not occurring over the course of our experiments and thus the decreases in inflammatory cytokine secretion and expression may be attributed to the presence of cromolyn and F-cromolyn.

**Figure 4:**
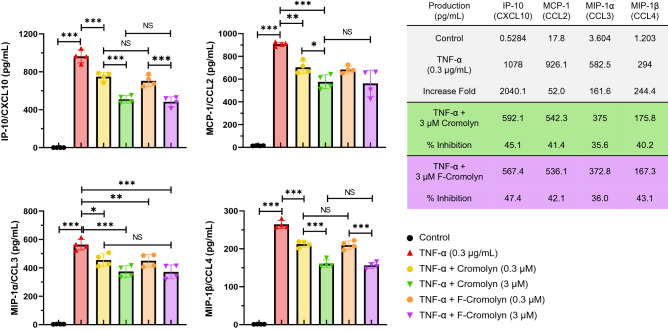
Cromolyn and F-Cromolyn inhibit the secretion of inflammatory chemokines induced by TNF-α in HMC3 microglia cell line. Quantitative analyses of MSD assays indicate the dramatically increased secretion of IP-10 (CXCL10), MCP-1 (CCL2), MIP-1α (CCL3) and MIP-1β (CCL4) from HMC3 cells treated with TNF α (0.3 µg/mL, 24 h), which are subsequently reduced by cromolyn and F-cromolyn (0.3 µM and 3 µM, each). *p < 0.05, **p < 0.01, ***p < 0.001, and NS (no significant difference).

We next tested the effect of cromolyn and F-cromolyn on the production of extracellular chemokines after the treatment of HMC3 cells with 0.3 µg/mL TNF-α (Fig. 4). Treatment with 0.3 µM cromolyn resulted in a statistically significant reduction in the concentration of extracellular IP-10 (CXCL10) (*p* < 0.001), MCP-1 (CCL2) (*p* = 0.0016), MIP-1α (CCL3) (*p* = 0.0118), and MIP-1β (CCL4) (*p* < 0.001) compared to cells treated with TNF-α alone for 24 h. Further, we observed reduction in extracellular chemokine secretion at a higher concentration of 3 µM cromolyn, with > 40% inhibition of IP-10 (CXCL10), MCP-1 (CCL2), and MIP-1β (CCL4), and 35.6% inhibition of MIP-1α (CCL3) compared to TNF-α cytokine induction levels.

Additionally, we observed that 0.3 µM F-cromolyn inhibited the production of inflammatory chemokines IP-10 (CXCL10) (*p* < 0.001), MCP-1 (CCL2) (*p* < 0.001), MIP-1α (CCL3) (*p* = 0.0081), and MIP-1β (CCL4) (*p* < 0.001). We also observed the higher concentration of 3 µM F-cromolyn impacted HMC3 microglia chemokine secretion, resulting in > 40% inhibition of IP-10 (CXCL10), MCP-1 (CCL2), and MIP-1β (CCL4), and 36.0% inhibition of MIP-1α (CCL3). Taken altogether, we conclude that only cromolyn dose-dependently inhibited MCP-1 (CCL2) and MIP-1α (CCL3) secretions but both cromolyn and F-cromolyn dose-dependently reduced secretions of IP-10 (CXCL10) and MIP-1β (CCL4) by HMC3 microglia cells.

**Figure 5:**
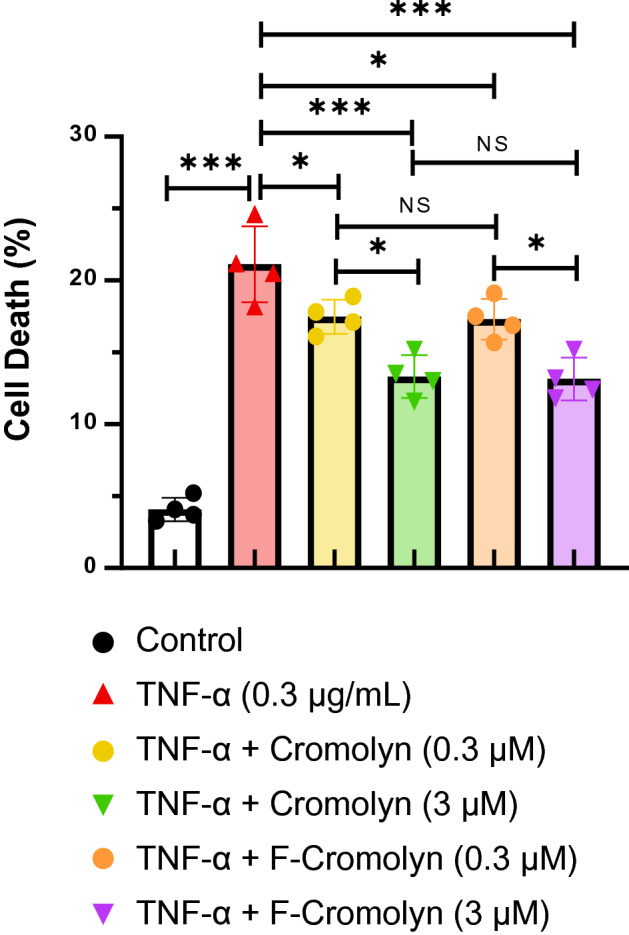
Cromolyn and F-Cromolyn reduced TNF-α induced cell death in the HMC3 microglia cell line. The comparisons of multiple groups were carried out by one-way ANOVA, followed by Bonferroni’s post-test. *p < 0.05, **p < 0.01, ***p < 0.001, and NS (no significant difference).

Since cromolyn and F-cromolyn were found to reduce concentrations of secreted neurotoxic cytokines and chemokines, we next carried out cell necrosis experiments to verify if this property translated to enhanced cell viability. Figure 5 shows that administration of TNF-α (0.3 µg/mL) to HMC3 cells was sufficient to increase cell death to approximately 20% of the total population, over four times that of controls. Addition of 0.3 µM cromolyn and F-cromolyn, respectively, significantly reduced cell death. Addition of higher 3 µM concentrations of cromolyn and F-cromolyn significantly reduced cell death even further, to increasing viability by approximately 40% over TNF-α induced HMC3 cells alone. We conclude that cromolyn and F-cromolyn are able to prevent HMC3 microglia cell death induced by TNF-α in a concentration-dependent manner.

## Discussion

Despite the robust cytokine and chemokine release by HMC3 microglia after addition of TNF-α, we observed several mediators that exhibited modest or no significant change in our experiments (Supplementary Figure S5). IL-2 is modestly expressed in HMC3 microglia after TNF-α exposure in our experiments and cromolyn appears to have no significant effect on the amount of IL-2 secretion. IL-2 is primarily produced by T cells and dendritic cells in the response to bacterial lipopolysaccharide (LPS) and is involved in T and B cell proliferation and differentiation^44^. Nuclear factor of activated T cells (NFAT) signaling mediates production of many pro-inflammatory mediators in dendritic cells, including IL-2 and TNF-α^44^. Although experiments with murine microglia have been shown to express NFAT isoforms, TNF-α does not significantly induce NFAT-driven luciferase activity in microglia as LPS does, suggesting that NFAT signaling in microglia may be more specifically induced than other peripheral immune cells^45^. Despite that microglia express IL-2 receptors and potently respond to exposure to IL-2 by activating expression of other cytokines^46^, TNF-α itself is not a significant inducer of IL-2 secretion by HMC3 microglia and cromolyn does not significantly change this behavior.

IL-10 is anti-inflammatory cytokine strongly produced by microglia in response to pathogen-associated molecular patterns (PAMPs) such as microbial lipopolysaccharides (LPS), against which the body is well known to initiate strong and lasting immune responses^47^. TNF-α has been found to induce IL-10 secretion alone in a dose-dependent manner in primary human fetal microglia over a 48-h exposure. However, our experiments with HMC3 microglia utilized 3 times the concentration of TNF-α (0.1 µg/mL vs. 0.3 µg/mL)^48^ and yielded approximately 6 orders of magnitude less the amount of IL-10 (Supplementary Figure S5). This result was surprising, but others have found that HMC3 microglia do not produce detectable amounts of IL-10 at rest^24^ as we can confirm. Neither cromolyn or F-cromolyn had any significant effect on altering IL-10 expression in HMC3 microglia. Additionally, in vivo experiments with the SOD1^G93A^ mice model of ALS found that 5 days per week intraperitoneal cromolyn treatment for approximately two months beginning 60 days postnatal until onset of major paralysis did not significantly alter IL-10 levels in spinal cord lysates despite mice exhibiting delayed onset of disease^23^. Future experiments will warrant exploring co-stimulatory factors to model anti-inflammatory IL-10 expression in this particular microglial cell line to further elucidate whether cromolyn or F-cromolyn has any effect on its secretion.

IL-4 is generally considered an anti-inflammatory cytokine when not chronically expressed that can downregulate the expression of pro-inflammatory mediators including TNF-α, IL-1β, IL-6, IL-8, and IL-12^49^. Though microglia are particularly responsive to exogenous IL-4 and the cytokine been associated with reducing expression of MHC-II, IL-4 is primarily produced by mast cells, basophils, eosinophils, and T helper cells^50^. As we observe little expression of IL-4 after administration of TNF-α to HMC3 microglia (Supplementary Figure S5), it is reasonable that IL-4 acts primarily on microglia to influence actions against neuroinflammation and that microglia do not appreciably secrete IL-4. Additionally, neither cromolyn or F-cromolyn significantly affected IL-4 secretion by HMC3 microglia induced by TNF-α.

CCL11 is a chemokine eosinophil attractant involved in allergic reactions and is found to be upregulated in blood sera and in the CNS in aging individuals^51^. Although microglia express CCR3 surface receptors for the CCL11 chemokine and produce neurotoxic reactive oxygen species in response to CCL11, it is activated astrocytes that have primarily been found to produce CCL11, specifically in the presence of 0.01 µg/mL TNF-α and not microglia^51^. Our experiments utilizing a much higher concentration (0.3 µg/mL) of TNF-α promoted detectable amounts of CCL11 (> 60 pg/mL) in HMC3 microglia but neither cromolyn nor F-cromolyn significantly altered their CCL11 secretion (Supplementary Figure S5).

As this study focused on the ability of cromolyn and F-cromolyn to prevent cytokine and chemokine secretion in HMC3 microglia, imminent experiments with RNA sequencing, proteomic binding assays, and biomarker panels of multiple cell types are needed to fully verify whether the mechanism of action of cromolyn to prevent cytokine secretion may be due to more broadly altering upstream/downstream cytokine gene transcription or by specific inhibition of cellular export either through exocytosis or secretion through the constitutive secretory pathway.

We have verified that HMC3 microglia exhibit robust inflammatory profiles when activated with TNF-α. Cromolyn and F-cromolyn are found to reduce the release of several key cytokines and chemokines related to neuroinflammation from TNF-α activated HMC3 microglia cells in a dose-dependent manner. Further, we found that cromolyn and F-cromolyn were able to prevent cell death in a dose-dependent manner compared to TNF-α activated controls. These results further expand our promising cromolyn platform for the potential treatment of neurodegenerative disorders to dampen the inflammatory profile of activated microglia.

## Supplementary Information

Supplementary Information.
