# Supplementary material for: Cromolyn inhibits the secretion of inflammatory cytokines by human microglia (HMC3)
Source: Sci Rep. 2021 Apr 13;11:8054. doi: 10.1038/s41598-021-85702-8 (PMC8044132; doi:10.1038/s41598-021-85702-8)
Supplement: Supplementary file 1 — Supplementary Information. [file 41598_2021_85702_MOESM1_ESM.docx]

**Supplementary Information for:**

**Cromolyn inhibits the secretion of inflammatory cytokines by human microglia (HMC3)**

**Yi-Jun Wang^1^, Alina Monteagudo^1^, Matthew A. Downey^1^, Philip G. Ashton-Rickardt^1^, and David R. Elmaleh^1,2*^**

Affiliations: ^1^AZTherapies Inc., Boston, MA, USA. ^2^Department of Radiology, Massachusetts General Hospital and Harvard Medical School, Boston, MA, 02129- 2060, USA. *email: delmaleh@mgh.harvard.edu

**Figure S1.** Activation by TNF-α and IFN-γ increase the percentage of positive cell population that express inflammatory microglia biomarkers in human microglia HMC3. Flow cytometric diagrams show the increased % of positive cells expressing inflammatory microglia biomarkers (MHC-II, CD68, CD14, IBA-1, CD40, CD86) in the HMC3 cells treated with IFN-γ (0.3 µg/mL, 24 hr), but no alteration in the % of positive cells expressing anti-inflammatory microglia biomarkers (CD163 and CD206). TNF-α (0.3 µg/mL, 24 hr) also increase the % of positive cells expressing inflammatory microglia biomarkers (CD14, IBA-1, CD40). We also include cytometric data for the observed increases in GFAP.

**Figure S2.** (a) Flow cytometric analyses demonstrate the increased protein expression of glial fibrillary acidic protein (GFAP) in the HMC3 cells treated with IFN-γ (0.3 µg/mL, 24 hr) (red) and TNF-α (0.3 µg/mL, 24 hr) (blue). (b) q-PCR analysis of GFAP mRNA expression increases relative to control after TNF-α (0.3 µg/mL, 24 hr) (blue) and IFN-γ (0.3 µg/mL, 24 hr) (red) administration to HMC3 microglia. ***p < 0.001 and *p < 0.1.


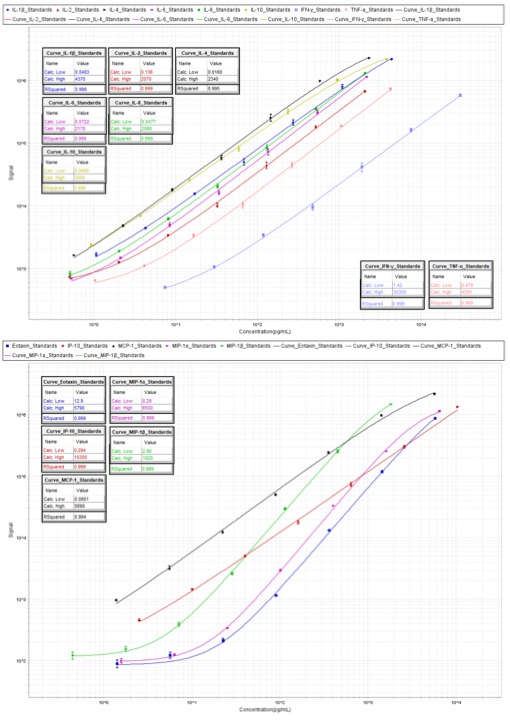


**Supplementary Figure S3.** Standard concentration curves of human inflammatory cytokines and chemokines.

**Figure S4.** Neither cromolyn nor F-cromolyn induced secretion of inflammatory cytokines or chemokines in human microglia HMC3.


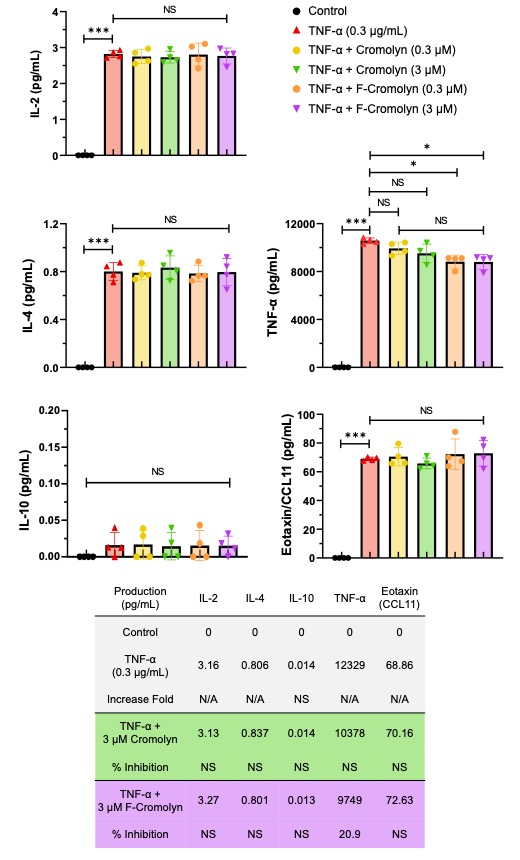


**Supplementary Figure S5.** Cromolyn and F-Cromolyn affects modest or no significant change in other inflammatory cytokine and chemokine secretions induced by TNF-α in HMC3 microglia cell line. *p < 0.05, **p < 0.01, ***p < 0.001, and NS (no significant difference).
